# Streamlined Fabrication and Acoustofluidic Purification of Silver-Decorated Polystyrene Microspheres (PS-AgNPs) for SERS Applications

**DOI:** 10.1021/acsanm.5c04981

**Published:** 2025-12-30

**Authors:** Jakub Novotny, Lucie Brezinova, Vit Pavelka, Anna Tycova

**Affiliations:** † 86852 Institute of Analytical Chemistry of the CAS, v.v.i., Brno 602 00, Czech Republic; ‡ Department of Chemistry, Masaryk University, Brno 625 00, Czech Republic

**Keywords:** acoustofluidics, immobilization, PS-AgNPs, polystyrene, silver nanoparticles, sorter, surface-enhanced Raman spectroscopy, wet etching

## Abstract

Composite microspheres of polystyrene and silver (PS-AgNPs) are highly valuable materials for catalysis, sensing, and antibacterial applications, yet their fabrication and subsequent purification remain challenging. This work presents a streamlined pathway for PS-AgNPs production, initiated by our finding that commercially available polystyrene (PS) microspheres (diameters ≥5 μm) anchor residual stabilizing polymeric structures that spontaneously facilitate the firm attachment of premade silver nanoparticles. The purified PS-AgNPs microspheres were evaluated as potential surface-enhanced Raman spectroscopy (SERS) substrates using adenine and thiamine as probe molecules, showing uniform SERS responses (coefficient of variation ≈ 10%) and limits of detection (LOD) of 100 nM and 1 μM, respectively. This demonstrates strong plasmonic activity that is suitable for sensing applications. While the synthetic approach is highly straightforward, it inherently creates a need to remove free and weakly attached nanoparticles, a critical step for PS-AgNPs practical application. We innovatively address this challenge by developing an acoustophoretic-based glass microfluidic device. Notably, the microchip was fabricated by using isotropic wet etching, a highly accessible method traditionally considered unsuitable for the precise geometries required for acoustophoresis. The separation principle relies on differential acoustophoretic migration, where larger PS-AgNPs microspheres are redirected into a collection outlet, while loose nanoparticles continue into the waste output, ensuring a high-purity final product.

## Graphical abstract

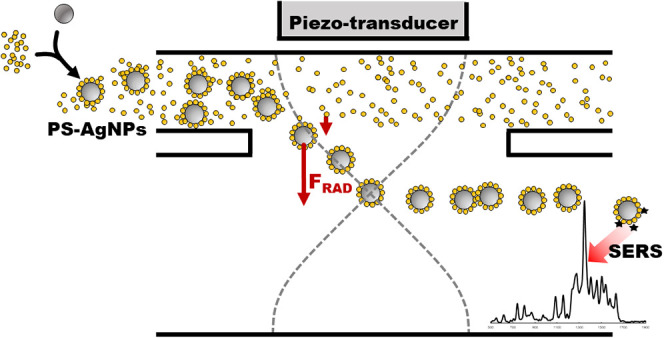

## 1 Introduction

Silver nanoparticles (AgNPs) are popular in bioanalytical chemistry for their plasmonic behavior, antibacterial properties, and catalytic effect.^1–4^ Among these, their ability to generate intense electromagnetic field enhancement under light excitation makes them exceptionally valuable for surface-enhanced Raman spectroscopy (SERS), enabling ultrasensitive molecular detection. These unique properties originate from their nanometric scale, which is, unfortunately, relatively simple to lose (e.g., by the inappropriate ionic strength of the solution).^5^ Although this can be effectively addressed by surface chemical modification of nanoparticles, this procedure results in chemical shielding of the silver surface and prevents direct contact with the investigated/treated solution. The immobilization of AgNPs onto a larger carrier is a powerful alternative to chemical treatment. This approach can facilitate the removal of unattached silver nanoparticles from a system and, in some cases, enhance material properties.^1,6,7^ The immobilization onto polystyrene (PS) latex microspheres is an interesting option, as this strategy preserves the character of the dispersion and, thus, compatibility with fluidic systems.

Additionally, PS microspheres are chemically inert and an easily accessible material. Unfortunately, the available literature indicates that anchoring silver nanostructures on polystyrene microspheres and forming PS-AgNPs are not a trivial task. The main strategy typically involves surface functionalization of the PS particles, followed by in situ chemical reduction of silver ions. PS microspheres are often prepared with specific functional groups on their surfaces (such as carboxyl, nitrile, or sulfonic) that provide a negative charge.^8–10^ Due to the electrostatic attraction of positively charged silver complex ions, silver is subsequently reduced directly on the PS surface. Alternatively, the trapping of in situ reduced AgNPs can be achieved via polymeric structures rich in amine/imine functional groups attached to the PS surface.^1,11–13^ Various reducing agents like citrate,^11,12^ NaBH_4_,^1,14,15^ or hydrazine hydrate^8^ were reported. Although some of the procedures reach a considerable level of silver decoration, the in situ reduction provides a very narrow optimal reaction window for the controlled formation of AgNPs. A significantly less common approach for obtaining PS-AgNPs relies on introducing a positive surface charge onto the PS spheres, which facilitates the electrostatic immobilization of preformed, negatively charged AgNPs.^16–18^ However, this strategy typically requires laborious in-lab synthesis of PS particles with well-defined surface properties, demanding a high level of expertise, extensive optimization, and handling of hazardous chemicals.

Residual reactants and/or free AgNPs staying freely dispersed in the liquid are another burning topic, which may challenge the PS-AgNPs applications.^17^ Therefore, purification of PS-AgNPs from the reaction mixture should be the final step in the synthesis process. Centrifugation is an effective and feasible method for separating particles from a solution based on their sedimentation speed. However, this technique allows only a portion of the supernatant to be replaced, and therefore, several centrifugation cycles are required to completely remove unattached nanoparticles and residual reactants, which extends the purification process. Moreover, in some cases, a high concentration of particles trapped in the pellet can impact the colloidal stability of the mixture.

Therefore, the use of flow-through microfluidic systems based on acoustofluidic principles has recently gained our attention as a promising approach for the efficient purification of colloidal materials. Acoustofluidics, a subfield of microfluidics, is a modern technique that utilizes the interaction of fluids and dispersed particles with ultrasonic waves in microfluidic channels. In this case, it allowed for particle manipulation via a pressure gradient generated by a sound wave. When a traveling sound wave meets an opposite or reflected wave of a matching wavelength, they will resonate while forming a so-called acoustic standing wave (ASW). The sound waves induce perturbations in the fluid that can manifest as several phenomena. Perturbations in fluid velocity manifest as a drag force called acoustic streaming. The pressure gradient between the antinodes and the nodes of the ASW manifests as the acoustic radiation force *F*_RAD_ (eq 1) and, in turn, as a particle migration phenomenon known as acoustophoresis.

1 $$F_{\mathrm{RAD}}=4\pi a^{3}\phi E_{\mathrm{AC}}\cdot \sin\left(2\mathrm{kx}\right)$$

where *a* is the size of the particle, *E*_AC_ is the energy density of the acoustic field, i.e., the acoustic energy per unit volume, *k* is the wavenumber, and *x* is the characteristic distance within the field.^19^

The acoustophysical properties of the particle within an acoustic field are defined by acoustic contrast factor ϕ. The value is dependent on the properties of both the medium (density, viscosity, compressibility, speed of sound) and the particle (size, density, compressibility). The difference in acoustophysical properties between microparticles is the basis for various label-free separation methods, such as particle sorting and acoustic trapping.

The radiation force is based solely on the mechanical properties of the system and is nearly independent of chemical properties such as pH, ionic strength, or charge, making it versatile enough to be applicable to even biological particles. The larger, denser, and less compressible particles are more susceptible to acoustophoretic migration because they are affected by a higher radiation force. By modifying the properties of the liquid medium, it is possible to alter the contrast factor of a particle and potentially widen the differences in the migration of previously inseparable particle populations.^20^

Because the formation of ASWs is imperative for the functionality of the acoustofluidic devices, the materials that support the geometry that could facilitate the reflection and resonation of sound waves are utilized in the fabrication of such devices, among others the rectangular-cross-section capillaries.^21–23^ For rigid microfabricated devices, the preferred material would be silicon, which can be dry-etched to form microchannels with flat, near-vertical walls. These relatively rectangular channels facilitate the resonance of sound waves because of the near-specular reflection. This is highly desirable because in microfluidic devices made from rigid materials, the microchannel itself acts as the resonator. The traveling wave is reflected by the wall back into the cavity and resonates with the incoming wave.^24–26^ Rounded or slanted walls could therefore be expected to be detrimental to this reflection and resonance. On the other hand, in microfluidic devices made out of soft materials, ASW forms by a resonance of sound waves from two opposing acoustic emitters.

This work introduces an extremely simple synthetic pathway for PS-AgNPs production and their flow-through acoustofluidics-based purification using a wet-etched glass microfluidic chip. We revealed that the commercially available PS microspheres with diameters ≥5 μm anchor residual polymeric structures on their surface, which spontaneously facilitate the firm attachment of premade AgNPs. Additionally, using the acoustophoretic effects of the ASW generated in a resonance chamber, the PS-AgNPs were continuously diverted from the crude reaction mixture to a clean medium, removing excessive and weakly bound AgNPs. This purification was conducted in a wet-etched glass device enclosed in a 3D-printed case, which allowed for user-friendly operation. Notably, we experimentally proved that the rounding effect of the isotropic wet etching is not as substantial as expected and does not impair the ASW resonance. By employing surface-enhanced Raman spectroscopy (SERS), we directly verified that the silver surface remains unshielded and highly accessible, confirming its suitability for plasmonic enhancement and demonstrating the immediate functionality of purified PS-AgNPs as active SERS substrates.

## 2 Experimental Section

### 2.1 Reagents and Materials

#### 2.1.1 Chemicals for the Synthesis of PS-AgNPs

Hydroxylamine hydrochloride (99.9% Sigma-Aldrich), sodium borohydride (NaBH_4_, 99.99%, Sigma-Aldrich), silver nitrate (≥99.8%, Lach-Ner), sodium citrate tribasic dihydrate (≥99.0%, Sigma-Aldrich), sodium hydroxide (98%, Penta), 5 μm microparticles based on polystyrene (solid content 10%, Supelco), and freshly deionized water (resistance 18.2 MΩ).

#### 2.1.2 Chemicals for the Sorter Fabrication

Sulfuric acid H_2_SO_4_ (90%, Penta), hydrogen peroxide H_2_O_2_ (30%, Penta), hydrofluoric acid HF (39%, Penta), gold 99.99% sputter target (Safina), and chromium 99.99% sputter target (Quorum Technologies); gold etchant (per 100 mL of solution): 2.5 g of iodine I_2_ (Lach-Ner) and 10 g of potassium iodide KI (Mach Chemikálie spol.); chromium etchant (per 100 mL of solution): 16.5 g of ceric ammonium nitrate (NH_4_)_2_[Ce­(NO_3_)_6_] (Sigma-Aldrich) and 4.2 mL of perchloric acid HClO_4_ (80%, Lachema); and photoresist Microposit S1805 G2 (DuPont de Nemours), developer Microposit MF-319 (DuPont de Nemours), borosilicate glass substrate BOROFLOAT 33 (Schott Technical Glass Solutions), and lead zirconium titanate piezoceramic plate Pz26 Navy I (Meggitt, now CTS Denmark) were utilized.

#### 2.1.3 Chemicals for the 3D-Printed Case

3D-printer photoresin FormLab Standard Clear (55–75% urethane dimethacrylate, 15–25% methacrylate monomers, >0.9% diphenyl (2,4,6-trimethyl benzoyl) phosphine oxide) (Formlabs), and isopropyl alcohol p.a. (Penta) were used.

### 2.2 Preparation and Characterization of PS-AgNPs Composite Particles

#### 2.2.1 Synthesis of AgNPs

We synthesized AgNPs using a widely spread protocol based on the reduction of silver cations from silver nitrate using hydroxylamine hydrochloride as a reductant.^27^ To the 3.74 mL mixture of 3.2 mM NaOH and 0.8 mM hydroxylamine was rapidly added 0.53 mL of a solution of 2 mM silver nitrate under vigorous stirring. The stirring was terminated after 15 min. To reach its stable conditions, the colloid ripened at least 24 h before its use. The colloid was stored at a laboratory temperature. The concentration of silver in the colloid was 27 μg/mL. The typical size of the AgNPs ranged from 25 to 50 nm.

In some experiments, the nanoparticles were concentrated via microcentrifuge (MiniSpin Plus, Eppendorf) using 10,000 RCF for 6 min. Various volumes were discarded to obtain the desired concentration of silver (discussed in detail later). The sediment with residual supernatant was homogenized by 5–10 s of sonication and short vortexing.

#### 2.2.2 Preparation of PS-AgNPs Composite

To remove any possible stabilizers, the volume of 50 μL of polystyrene microspheres was dispersed in 950 μL of water and centrifuged for 6 min at 10,000 RCF (Mikro 22, Hettich). The volume of 900 μL of supernatant was discarded and replaced with fresh deionized water. The process of centrifugation and redispersion was then repeated twice; however, no refill was done in the final step. Thus, the PS microspheres were diluted 2×.

The volume of 10 μL of purified PS microspheres was mixed with 990 μL of AgNPs. The AgNPs did not undergo any special chemical pretreatment. The mixture of PS and AgNPs was continuously stirred for 5 min. Finally, the dispersion was stored at least for 30 min at laboratory temperature prior to its use. Due to spontaneous sedimentation, the PS-AgNPs dispersion was briefly vortexed and sonicated before each use. The hypothesized mechanism of PS interaction with AgNP is depicted in Figure 1.

**Figure 1:**
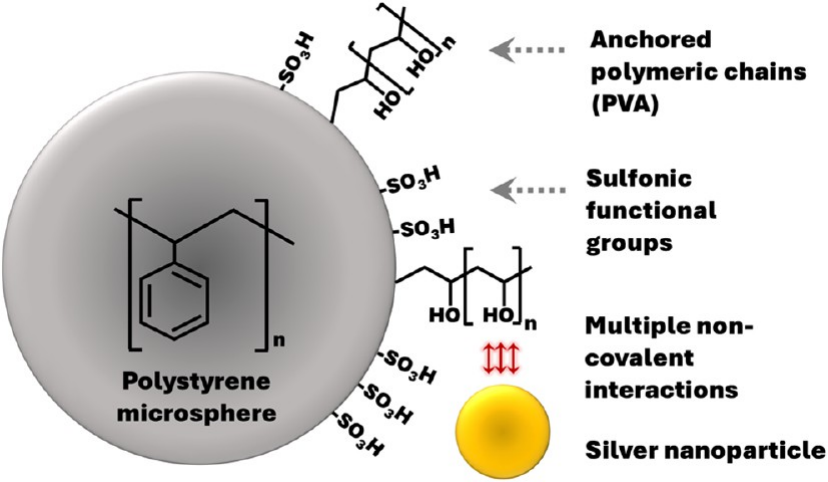
Schematic mechanism of AgNPs’ spontaneous attachment to 5 μm PS microspheres. The figure is not in scale.

#### 2.2.3 Effect of Poly­(vinyl alcohol) (PVA) on the Trapping of AgNPs

The experiment tested the effect of 0.2% solution PVA (205,000 g/mol, Sigma-Aldrich) on the trapping efficiency of AgNPs on the PS microspheres with the following diameters: 0.5 μm (solid content 2%, Supelco), 1 μm (solid content 2%, Supelco), 2 μm (solid content 2%, Supelco), 5 μm (solid content 10%, Supelco), 8 μm (solid content 2%, Sigma-Aldrich), and 10 μm (solid content 10%, Sigma-Aldrich).

The volume of 50 μL of PS microsphere dispersion was added to 950 μL of 0.2% PVA solution. The dispersion was left overnight on a rotator. In the second batch, a fresh 50 μL of PS microspheres was dispersed in 950 μL of ultrapure water and left on the rotator overnight as well. After the incubation, both the PVA-involved and PVA-free dispersions were centrifuged for 6 min using 10,000 RCF (Mikro 22, Hettich). The supernatant of 900 μL volume was discarded and replaced with 900 μL of ultrapure water. The same centrifugation procedure was repeated 3×. However, in a final centrifugation step, no refill with water was conducted. Thus, the original dispersion became diluted 2×.

The volume of 10 μL of the purified PS dispersions was mixed with 990 μL of 5× concentrated AgNPs reduced by hydroxylamine hydrochloride (i.e., 135 μg/mL of silver) and let on a rotator for 3 h. Then, the dispersions were inspected by scanning electron microscopy (SEM).

#### 2.2.4 Electron Microscopy

The PS-AgNPs particles were inspected by scanning electron microscopy (Mira3, Tescan). The samples were dropped on a piece of silicon wafer and left to dry freely at laboratory temperature. For the collection of the pictures, the acceleration voltage was set on 10 kV with a beam intensity of 12 eV. The picture was created by the combination of signals from the InBeam (90%) and SE (10%) detectors. No conductive coating was applied to the samples.

#### 2.2.5 Measurement of ζ-Potential

Dynamic light scattering (Zetasizer Nano ZS, Malvern) with a disposable folded capillary cell (DTS1070) was used for ζ-potential measurements. A temperature of 25 °C was equilibrated for 2 min. Three repeated measurements and the automatic duration of the measurement were applied.

#### 2.2.6 Surface-Enhanced Raman Spectroscopy

The surface chemistry and plasmonic features of PS-AgNPs microspheres were inspected using a Renishaw inVia Reflex confocal Raman spectrometer. Unpolarized backscattered Raman spectra were acquired using the 1800 l/mm grating and two diode lasers with wavelengths of 633 and 532 nm and powers of 33 and 50 mW, respectively. For both conventional Raman and mapping measurements, ×50 Nikon TU Plan ELWD with NA 0.60 and a Renishaw CCD Centrus 1024 × 256 detector were used. Conventional Raman spectra were acquired using a 10 s acquisition time and 532 nm excitation wavelength. Large-scale SERS maps were acquired using a step of 1.0 μm, 100 ms acquisition per point, and 633 nm excitation wavelength. The nominal signal of laser power during mapping was set to 1%, resulting in 110 μW of laser power at the point of measurement. Adenine (≥99%, Sigma-Aldrich) and thiamine hydrochloride (≥99%, Sigma-Aldrich) were chosen as the SERS reporters. Solutions of various concentrations were prepared from both analytes. 4 μL of a solution was mixed with 4 μL of PS-AgNPs and vortexed for 10 s, then transferred in the form of 0.5 μL droplets onto a silicon wafer. The Raman and SERS spectra were collected from dried spots of the sample after focusing on the silicon wafer and fixing the Z-position at 5 μm. All spectra were processed via an automated pipeline based on morphological filtering described elsewhere.^28^

### 2.3 Fabrication of the Acoustofluidic Sorter

#### 2.3.1 Photolithography

The borosilicate glass substrates were properly cleaned with detergent and water and then with isopropanol. The cleaned substrates were left in piranha solution (3 parts sulfuric acid and 1 part hydrogen peroxide) overnight to remove all remaining traces of organic impurities. The substrates were desiccated on a hot plate and coated with 80 nm of chromium and 60 nm of gold using sputter coating (Quorum Q300T D). The substrate was then spin-coated with positive photoresist Microposit S1805 G2 at 3000 rpm for 30 s, resulting in a 5 μm thick layer. The patterns of the microchannels were transferred to the photoresist by the direct pattern writer MicroWriter ML3 from Quantum Design. The exposed resist was developed, and the patterns were etched into the metal layers using etchants of potassium iodide/iodine solution for gold and ceric ammonium nitrate/perchloric acid solution for chromium. The processed glass substrates were then exposed to the glass etchant.

#### 2.3.2 Glass Etching

The glass etchant was mixed from 3 parts of hydrofluoric acid, 1 part of sulfuric acid, and 4 parts of deionized water. While the HF etched the glass by reacting with the molecules of silicon oxide, H_2_SO_4_ dissolved byproducts of the reaction, which could obstruct the etching process. For the etching procedure, the glass etchant was placed into a Teflon dish and tempered to 50 °C.

The device was designed as 2 matching mirrored units. The etching depth of 40 μm was indicated using depth gauge structures. The design of the microfluidic system contained one 100 μm inlet (primary) channel and one 250 μm (secondary) inlet channel that converged in a 400 μm wide resonance (separation) chamber. The chamber then split into the outlet channels that mirrored the inlets, a 100 μm primary and a 250 μm secondary outlet channel. The primary and secondary channels were separated by a 50 μm divider. The bonding of the complementary mirror units resulted in closed channels with a depth of 80 μm. The layout of the sorter is given in Figure 2A.

**Figure 2:**
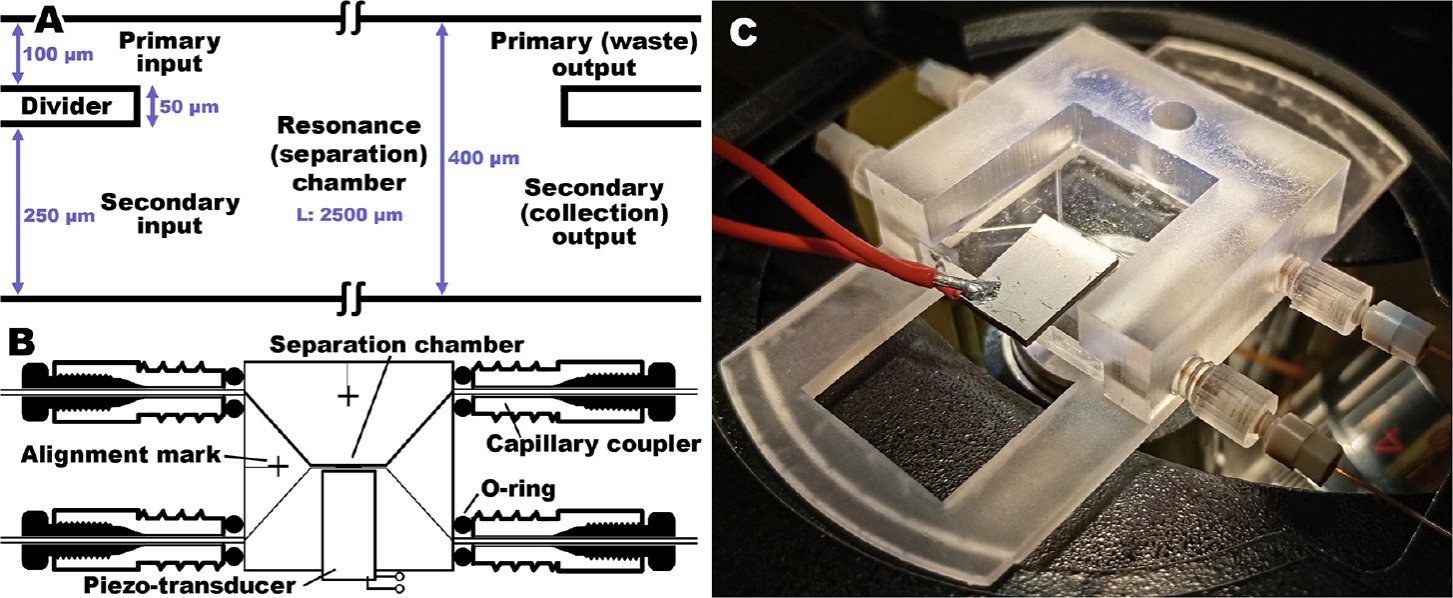
(A) Design and dimensions of key parts of the acoustophoretic sorter. (B) Diagram of the chip and the connections. The o-rings tightened around the openings of the chip by the screw-in couplers, in combination with tight fitted connections for the PEEK fittings and the narrow-bore ducts for the capillaries, ensured reliably sealed fluidic connections. (C) 3D-printed chip holder consisting of the frame and the screw-in couplers. The couplers are compatible with commercial PEEK fittings for 380 μm OD capillaries by LabSmith.

#### 2.3.3 Glass Bonding and Final Shaping

Precisely aligned glass pieces were sandwiched between finely polished ceramic wafers and plates of heat-resistant glass, all dusted with a thin layer of graphite powder to prevent undesired fusions. Set under 2.2 kg of weight, the glass pieces were bonded in a furnace at 600–630 °C. The heating program was set to slowly ramp to 100 °C for an hour and then kept at 100 °C for an hour to release all possible water adsorbed to the glass or trapped between the pieces. After the desiccation step, the temperature was ramped for 50 min to the bonding temperature, which was then kept for 10 h. After the end of the program, the glass was kept in the furnace until it naturally cooled to room temperature to reduce any possible stress within the glass. Because the input and output openings were situated on the sides of the chip, these had to be released by cutting the chips to the final shape of 20 mm × 20 mm on a diamond CNC dicing saw (MTI SYJ-800).

#### 2.3.4 3D-Printed Frame and Fluidic Connections

A 3D-printable holder was printed using a FormLab Form3 stereolithography (SLA) printer. The holder frame could fit into a microscope mount for round wafers (65 mm diameter) and used up only 7 mL of the printing resin. The glass chip of 20 mm × 20 mm was inserted into a slot in the frame and secured by NBR o-rings tightened around the inlets and outlets by 4 screw-in couplers compatible with the PEEK fittings for fused silica capillary (LabSmith Inc.) (Figure 2B,C).

#### 2.3.5 Acoustics

A piece of PZT (lead zirconium titanate) piezoceramic plate Pz26 Navy I was attached to the glass with cyanoacrylate superglue, 2–3 mm away from the channel. The acoustophoretics operated on the principle of a half-wavelength resonator. It is a design where the sound wave propagating in a chamber resonates with a reflected wave to form the ASW when the width of the chamber is an integer multiple of the half-wavelength of the waves. Based on the simple calculation using eq 2 for our 400 μm resonator

2 $$f=n\frac{c_{f}}{2w}$$

in which *w* is the width of the channel, *n* is the number of nodes in the ASW, and *c*_*f*_ is the speed of sound in the medium (i.e., about 1500 m/s in water), a single-node ASW would be theoretically generated at a frequency of 1.875 MHz.

The piezoceramics were powered by a sine-wave produced by an arbitrary wave function generator (OWON XDG3102). The maximum voltage amplitude generated by the generator was 10 V_PP_, although this had to be optimized either to smaller input voltage amplitudes or to an amplified voltage, vice versa, to facilitate the separation of particles. For instance, applying an acoustic field of 6 V_PP_ was sufficient to deflect PS-AgNPs 5 μm microspheres.

The stability and service life of the piezoceramic plates were never an issue during regular operation, but repeated reuse, i.e., removal from discarded devices using organic solvents, regluing to new devices, and resoldering of the wires, could damage the conductive layers of the surface, rendering the plates inoperable.

Under real conditions, though, where the etching could not be stopped at precisely 40 μm and the size of the resonator varied slightly between chips, the frequency required tuning for each glass device. The optimization involved a slow sweep through a set range, increasing the frequency by kHz every few seconds. As it was not an available preset function in the wave function generator, a LabVIEW VI was created to construct strings of SCPI-code commands for the generator based on the numerical values and dial settings input by the operator. The optimization process was captured by a microscope camera (Kern ODC 862) and the optimal frequency determined from the footage.

The general operation of the device was as follows: The mixture of particles was pumped through the primary inlet, while a clean medium was pumped through the secondary inlet. When the ASW was off, the laminar flow and the suppressive stream of the clean medium would force the particles to stay in the straight trajectory and enter only the primary outlet. The ratio of flow rates was generally 1:3–1:4, which seemed sufficient to exclude any particles from spontaneously entering the secondary outlet. The ASW would force the particles of higher acoustophoretic mobility to overcome this suppressive drag and enter the wider secondary outlet (Figure 3). The initial proof-of-concept experiments were conducted on mixtures of PS microspheres with various diameters ranging from 0.5 to 12 μm, which confirmed the functionality of the device.

**Figure 3:**
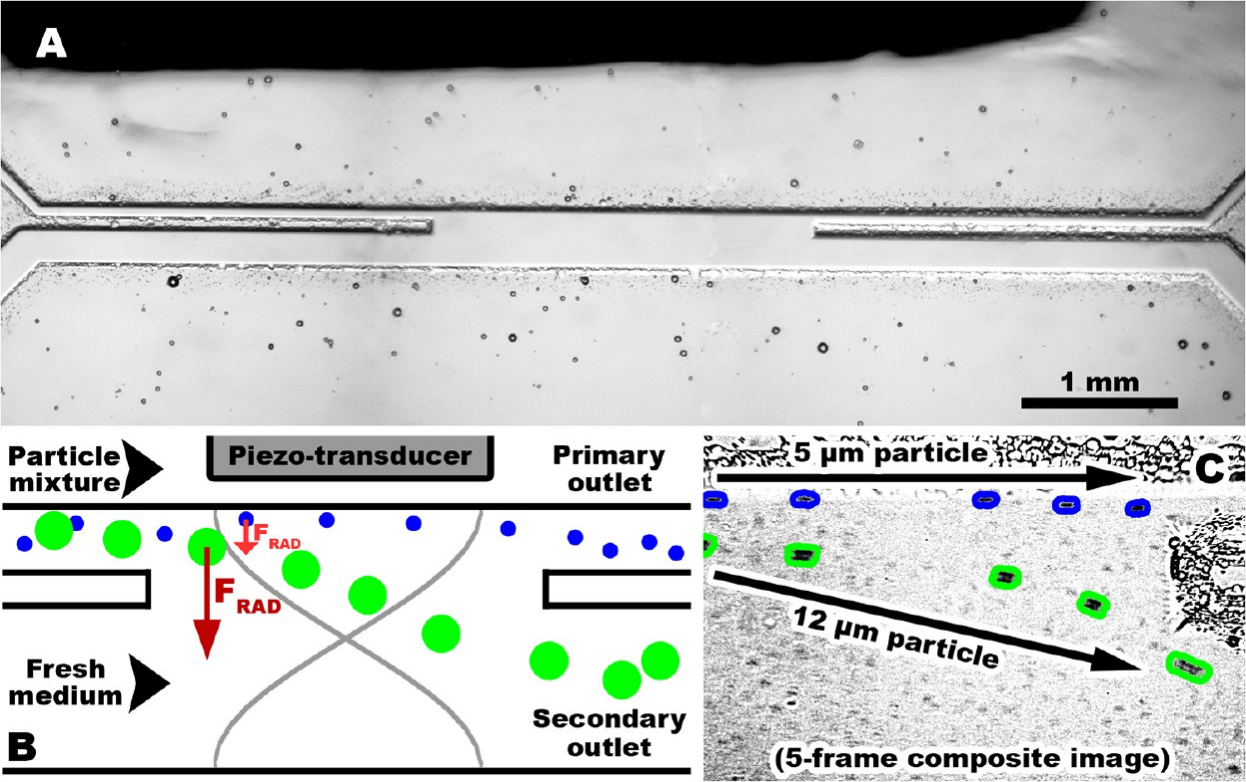
(A) Photograph of the etched-glass microchannel showing the arrangement of the separation chamber. (B) Diagram of the separation chamber and functionality of the chip. (C) Composite photo of 5 frames of a video taken while applying an acoustic field to the separation chamber, showing trajectories of particles of 2 different sizes (5 and 12 μm). Smaller particles *(highlighted blue)* stay on the straight trajectory, while larger particles *(highlighted green)* are diverged to the alternate secondary outlet by much higher radiation force.

The mixture was flown through the sorter device at a slow flow rate of 50–100 μL/h. The suppressive stream of the clear medium had a flow rate of 200–300 μL/h. The deflected PS-AgNPs microspheres could be collected at the secondary outlet. The fraction from the primary outlet of the continuous stream of sample was also collected, and the functionality of the device was confirmed by comparing the fractions from the primary and the secondary outlet using SEM.

## 3 Results and Discussion

The primary motivation for this study was the user-friendly and efficient synthesis of PS-AgNPs microspheres, a material with significant potential in catalysis, antibacterial applications, and, most importantly, surface-enhanced Raman spectroscopy (SERS). Therefore, we aimed to preserve the bare surface of silver, intentionally avoiding its covalent modification to maximize its intrinsic properties. The surface shielding is particularly detrimental in SERS applications, where direct analyte–silver contact is essential for achieving strong signal enhancement. Furthermore, we targeted obtaining the composite structure, which is free from excessive and weakly bound AgNPs, which might complicate future application of this material.

### 3.1 Interaction of AgNPs with PS Microspheres

In the synthesis of PS-AgNPs, our initial investigation focused on the spontaneous interaction between AgNPs, reduced by hydroxylamine hydrochloride, and commercially available 5 μm PS microspheres. According to the manufacturer, these microspheres carry on their surface sulfonic functional groups (−SO_3_H) introduced by persulfate during polymerization. As the ζ-potential of both microspheres (−38 mV) and AgNPs (−42 mV) is negative, their affinity is generally regarded as weak and insufficient to provide robust immobilization.^29^

However, contrary to expectations, scanning electron microscopy (SEM) analysis revealed a spontaneous decoration of the PS microspheres with AgNPs (Figure 4). The use of crude colloid (≈27 μg/mL of silver) resulted in a moderate surface coverage. Additionally, the decoration improved significantly with an increasing AgNPs/PS ratio, ranging from a uniform and compact monolayer to a massive multilayer. To investigate the strength of the binding, we transferred PS-AgNPs into deionized water and inspected the surface by SEM. Interestingly, the multilayers were during the washing completely released, while the AgNPs, being in contact with the PS surface, remained unaffected even after 12 days of storage (Figure S1).

**Figure 4:**
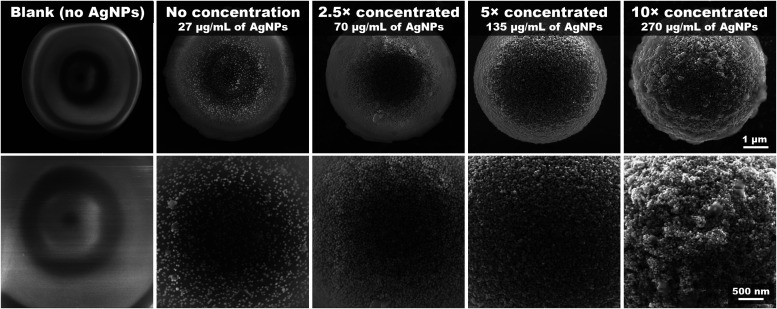
SEM figures depicting the change in the density of AgNPs on the surface of the polystyrene microspheres at two different magnifications. The concentration of AgNPs varied across the samples, as indicated in the figure, while the concentration of polystyrene microspheres remained constant.

This highly efficient attachment was unexpected and pointed toward a binding mechanism more potent than the anticipated AgNPs-SO_3_H interaction. We hypothesized that the origin of this phenomenon lay in the manufacturing process of the PS microspheres. In large-scale production, there are three main synthetic strategies all based on polymerization of styrene monomers driven by an initiator: (1) emulsion polymerization, (2) seed growth polymerization, and (3) dispersion polymerization.^30^ The first method deals with polymerization inside the micelles, of which the volumes determine the diameter of the produced microspheres. Thus, the *emulsion polymerization* is particularly useful for the synthesis of submicrometric spheres. To produce larger PS bodies, *seeded growth polymerization* can be used, which controls polymerization on the surface of smaller seeds synthesized in the emulsion. Additionally, *dispersion polymerization* is another powerful method for the micrometer-sized PS spheres. Styrene and initiator are dissolved in a solution and by the temperature-controlled reaction gradually form PS microspheres. Stabilizing structures play a crucial role in the PS microspheres preparations, as well as preventing coagulation among the particles in the formation stage. While emulsion and seeded growth polymerization operate with electrostatic repulsions from surfactants, the dispersion polymerization also employs polymeric structures—e.g., poly­(vinyl alcohol) (PVA) or polyvinylpyrrolidone (PVP)—acting as steric barriers. Importantly, some portion of stabilizers incorporates into the PS surface.^31^ We further propose that these distinct synthetic routes inherently influence the nanoscale surface roughness of the resulting PS microspheres, a trend that we observed in our SEM and AFM analyses (Figure S2).

Although the exact composition of stabilizing structures is not available publicly, the presence of PVA was confirmed by correspondence with the producer and was also confirmed experimentally by Raman measurements of the PS-AgNPs surface. While bare PS microspheres provide only a strong signal of polystyrene, the surface enhancement caused by AgNPs attached to the PS surface via PVA chains intensified its response, resulting in a characteristic signal of −OH vibration (3400–3600 cm^–1^) in spectra (Figure S3).^32^

We deduced that the 5 μm PS microspheres used for the PS-AgNPs synthesis were prepared by dispersion polymerization, and thus, these spheres carry on their surface residual stabilizing chains, which cannot be removed by washing procedures. Thereby, they might mediate the attachment of AgNPs. Indeed, PVA and similar polymeric structures are commonly used for silver colloid stabilization due to PVA-AgNP affinity, which arises from multiple noncovalent interactions, predominantly involving the hydroxyl groups of PVA.^33^

To verify this, we conducted a control experiment. PS microspheres with sulfonyl functional groups in the range of 10–0.5 μm in diameter were subjected to the AgNP colloid. Parallelly, we investigated their interaction with AgNPs, however, after their previous exposure to a 0.2% PVA solution. The microspheres with a diameter of ≤2 μm remained bare unless their surface was exposed to PVA solution (Figure 5). However, the microspheres with larger diameters were also decorated without any intentionally added PVA. This behavior was thoroughly tested on microspheres produced by four different producers (see Figure S4). This confirms that residual PVA on the surface of microspheres is the key enabler for the spontaneous and robust assembly of AgNPs on the larger (i.e., ≥5 μm) PS microspheres and highlights the enormous impact on the synthetic pathways on the PS-AgNPs production.

**Figure 5:**
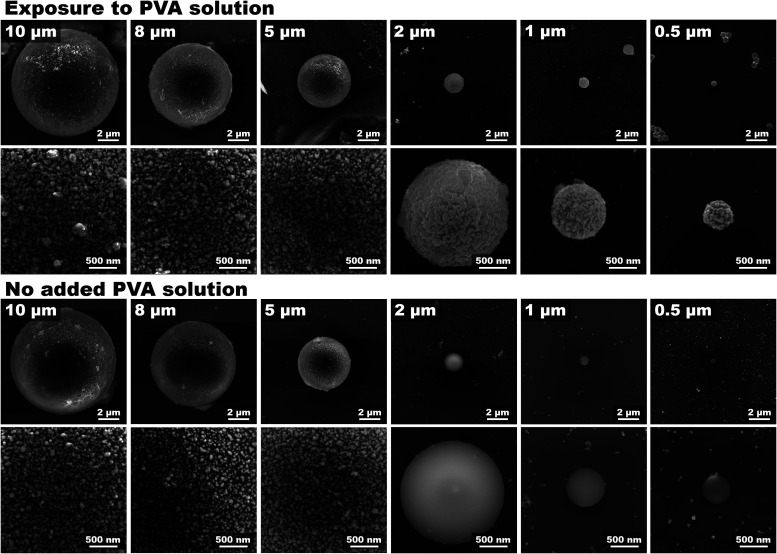
SEM figures of polystyrene microspheres (diameters in the range of 10–0.5 μm) exposed to AgNPs. The figure shows the effect of PVA solution on the surface coverage of PS microspheres.

The finding that incorporated polymer chains facilitate AgNP immobilization is extremely advantageous for PS-AgNPs preparation. This is because the silver nanoparticle surface remains largely exposed, which aligns with our primary objective in the design of this material. In contrast, simply mixing nanoparticles into a PS dispersion containing PVA would likely lead to complete surface coverage of the AgNPs by PVA molecules, thereby blocking access to the silver surface.

It should be stressed that this revealed spontaneous interaction of microspheres and AgNPs allows to avoid in situ reduction of silver, which is a prevalent procedure for previously published works on PS-AgNPs synthesis. This is a highly important aspect, as the in situ silver reduction significantly limits the properties of the reaction mixture and affects the quality of the product. Indeed, we compared the in situ synthesis of PS-AgNPs with our introduced strategy, decoupling the reduction and immobilization parts. Even though the in situ reduction resulted in the attachment of AgNPs to the PS surface as well, the surface coverage was influenced by the concentration of silver mildly (Figure S5), being at a moderate level. Additionally, the stability of the PS-AgNPs dispersion at increased silver concentrations quickly collapsed, and noticeable clusters of PS-AgNPs formed. The clustering was apparent to the naked eye through sedimentation (Figure S6).

To understand the reason for clustering, we measured the UV–vis absorption spectra of AgNPs synthesized at different concentration levels (Table S1). Interestingly, the spectra showed the stability of the silver dispersion even for a 5× increase of the concentration (Figure S7). However, the stability of PS-AgNPs dispersion is already affected at the lowest concentration factor. We deduced that the instability of the dispersions can have its origin in the overall increase of ionic strength of the mixture.

Additionally, we investigated the spontaneous affinity of AgNPs to the PS surface using two distinct and widely used synthesis protocols, citrate and sodium borohydride (NaBH_4_) reduction. We chose them intentionally because they produce nanoparticles of different sizes and have various capping agents. Citrate reduction, which requires boiling of the reaction mixture for tens of minutes, typically yields larger AgNPs (40–100 nm). In contrast, NaBH_4_ reduction is a rapid, low-temperature process that results in particles of several nanometers in diameter. The details of the synthesis are given in the Supporting Information.

In both cases, the AgNPs exhibited a strong spontaneous affinity to the PS microsphere surface, achieving excellent surface coverage (Figure 6), which is tunable by the AgNPs/PS ratio (Figure S8). This demonstrates that PS microspheres can effectively trap AgNPs regardless of their size or the used capping agent. This is an important observation, as it shows that the roughness of the silver layer can be readily tailored simply by choosing the appropriate type of silver nanoparticles. Although surface chemistry remains a key factor, it can be hypothesized that surface-dependent uses (such as catalytic or antibacterial applications) may benefit more from the large surface area of nanometer silver produced by NaBH_4_ reduction. In contrast, for SERS applications, larger silver nanoparticles—typically obtained via citrate or hydroxylamine reduction—are generally preferred due to their more favorable plasmonic properties.

**Figure 6:**
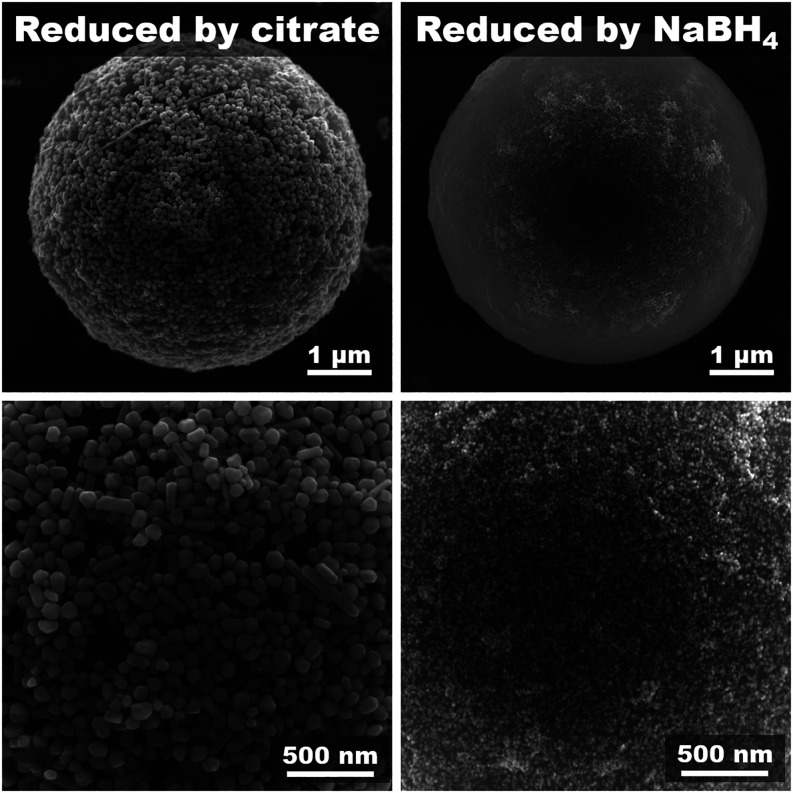
SEM Figures of PS microspheres decorated with AgNPs. The AgNPs were reduced by sodium citrate and sodium borohydride, using concentrations of AgNPs of 1080 and 70 μg/mL, respectively.

### 3.2 Application of PS-AgNPs as a SERS Substrate

The primary motivation of this study is to develop a strategy for stabilizing AgNPs in order to preserve their unique properties, which are often compromised by the loss of their nanostructured character. One of the most important bioanalytical applications that benefits from the plasmonic properties of nanoscale silver is surface-enhanced Raman spectroscopy. To eliminate free and weakly attached AgNPs, which could bias SERS measurements, the PS-AgNPs were purified by repeated centrifugation prior to the SERS measurements.

To evaluate the performance of the synthesized PS-AgNPs microspheres as SERS-active materials, we conducted measurements with adenine (a recommended SERS benchmark molecule) and thiamine using PS-AgNPs with 135 μg/mL of silver in the reaction mixture.^34^ First, we evaluated the signal uniformity, a parameter highly dependent on the homogeneity of AgNPs coverage, by integrating the SERS response of individual decorated microspheres within each map (Figure 7). Before the calculation of coefficient of variation (CV), we removed outliers based on the interquartile range of the integrated responses distribution. For adenine (500 nM), the CV was determined to be 12%, whereas thiamine (1 μM) exhibited a lower CV of 6%. These CV values are comparable to those reported for typical SERS substrates, where values below 10–20% are generally taken as indicative of good SERS substrate uniformity and reproducibility.^35,36^

**Figure 7:**
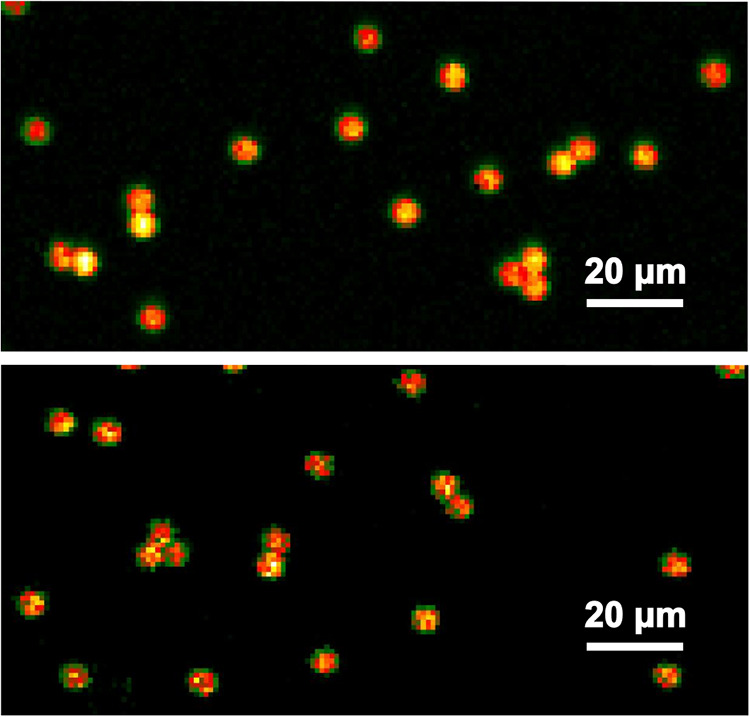
Large-scale SERS maps of dried PS-AgNPs microspheres mixed with SERS probes. Each pixel represents the Raman response of the probe’s fingerpeak, with a resolution of 1 μm and color-coded on a black-green-red-orange-yellow-white color scale. Top: 500 nM adenine (732 cm^–1^). Bottom: 1 μM thiamine (756 cm^–1^).

This is in drastic contrast to a CV value obtained if PS-AgNPs with 27 μg/mL silver in the reaction mixture were used. As these reaction conditions resulted in a much sparser AgNPs distribution on the microsphere surfaces (see also Figure 4), the CV for adenine (500 nM) was determined on 47%, which suggests that an insufficient and inhomogeneous net of electromagnetic hotspots on the microsphere surface strongly compromises control over SERS enhancement.

Second, we focus on the determination of the limits of detection (LOD) for both model analytes. The concentrations of their solutions were decreased stepwise, acquiring SERS maps under identical conditions. All spectra were filtered and aggregated. The LOD was defined as the lowest concentration at which a fingerpeak reached a signal intensity at least three times higher than the standard deviation of the spectral noise (*S*/*N* ≥ 3). Notably, both analytes provided fingerpeaks in spectral regions with minimal interference with the blank PS-AgNP spectrum.

According to this criterion, we obtained LOD values of 100 nM and 1 μM for adenine and thiamine, respectively (Figure 8). This nicely meets the values that are reported for polymer-supported, nanoparticle-decorated SERS substrates based on microspheres, which typically exhibit LODs in the 10^–7^–10^–9^ M range for low-molecular analytes.^37,38^

**Figure 8:**
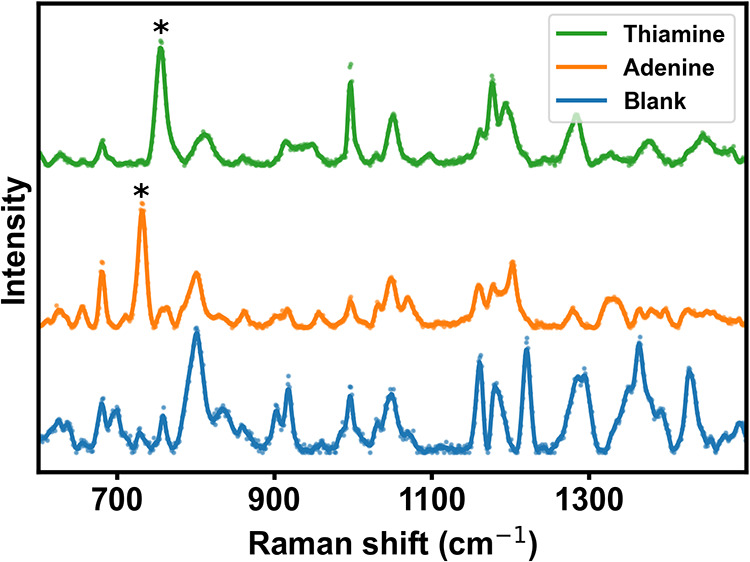
Filtered and aggregated SERS spectra of 100 nM adenine (fingerpeak at 731 cm^–1^) and 1 μM thiamine (fingerpeak at 755 cm^–1^) obtained from mapping experiments. Blank corresponds to PS-AgNP without an analyte. Asterisks label the positions of peaks used for LOD determination. Spectra are normalized and vertically offset for clarity.

It should be noted that the determined RSD and LOD values remained consistent over a week of measurements when the PS-AgNPs microspheres were stored in a refrigerator. This indicates good stability of both the AgNP coating and the underlying PS support.

Overall, Raman mapping experiments demonstrate that the developed optimized PS-AgNPs composite behaves as a promising, uniform, and stable SERS substrate. Prior to SERS measurements, we purified the dispersion by centrifugation, which serves as a powerful technique for processing large-volume samples. However, due to its operational principle, centrifugation is inherently incompatible with fluidic systems—platforms that are of high practical relevance for real-world analytical and sensing applications.^39–41^

### 3.3 Practical Considerations in the Chip Construction

A flow-through microfluidic device based on acoustofluidic manipulation of PS-AgNPs was developed to address the need for dispersion flow-through purification. The device could be classified among the so-called split-flow thin-film (SPLITT) separators. The design was inspired by works of Giddings,^42^ who proposed a gravitational-force-based separator of similar design, and by Johnson and Feke,^43^ who presented a millimeter-scale acoustic SPLITT separator. This general design, although based on some of the earliest publications on the topic of SPLITTs, was determined to be very suitable for microfabrication in glass by using wet etching.

Indeed, the microchannels were fabricated by using wet etching in HF. This method of etching is isotropic, meaning that the rate of etching is equal in every direction. As such, it produces structures with rounded corners and edges, and it is practically impossible to prepare channels with a sharp-edged cross section in glass. This is in contrast to the anisotropic dry etching, in which the etching progresses at a much higher rate along a specific direction.

This rounding effect was initially thought to be detrimental to the formation of ASWs because the proper reflection of the sound waves is essential in a half-wavelength acoustic resonator, such as our separation chamber. The design of our resonator was based on a transversal resonator. The operational principle of such a resonator would suggest that the ASW would form most efficiently if the reflecting walls are perfectly parallel, such as in the case of deep dry-etching methods for silicon.^44^ It was actually confirmed that glass-made acoustic resonators could show similar performance to the silicon resonators, despite their nonrectangular cross section.^45^

Theory behind the wet-etching process in glass would suggest that because it is progressing in all directions at the same rate, the profile of the channel after isotropic etching would be very rounded, almost U-shaped, while the directionally uneven anisotropic etching would leave a trapezoidal profile with (under optimal conditions) almost vertical walls. Profilometric analysis of several of our microchannels (before bonding) showed that the channel profile of these HF-etched glass channels was nowhere near as rounded as expected. The actual profile was a trapezoid with mildly rounded corners. The resulting bonded channels had the cross section resembling approximately an elongated hexagon (Figure S9). Because we were repeatably able to achieve acoustophoretic migration in these channels, it could be surmised that the geometry of our resonator was sufficient in reflecting the sound waves and sustaining the ASW.

In the initial design with the inlet and outlet openings situated on the surface of the chip, these had to be drilled into one of the glass pieces by using a diamond dental burr (Figure S10). The design was then changed to a smaller chip with in/outlets on the sides. This modification was motivated mainly by the fact that the side openings are significantly less prone to clogging. Indeed, as the chip is fabricated from the borosilicate glass, it is chemically extremely stable, and its long-term operations are limited mostly by the accessibility of the chip’s channels. On the contrary, the positions of the openings on the side of the chip required a high-precision CNC dicer to cut the sides with very even and smooth unchipped surfaces. Another design optimization aimed at the geometry of a divider (Figure S11).

The 3D-printed holders for the cut-out side-loaded chips provided quick-release fluidic connections and were mount compatible with microscope stage frames. The holders could be designed very economically and therefore required only 7 mL of printing resin (and an additional 5 mL for print supports).

### 3.4 Particle Sorting

Because our depth gauge system was rather arbitrary, depending purely on visual assessment, the channel dimensions never matched the theory exactly. Because of that, the focusing frequency needed to be optimized for each glass chip. While the theoretical ASW frequency for the resonance chamber was 1.875 MHz, the frequency sweep determined that the real ASW frequency for individual chips usually fell into a slightly lower range between 1.70 and 1.85 MHz.

The sorter required tuning of the ASW voltage amplitude depending on the flow rate. At lower flow rates (tens of μL/h), the maximum amplitude available with our wave function generator (10 V_PP_) was capable to also deflect a portion of the lower mobility particles into the secondary outlet. It was found that amplitudes of 6 V_PP_ and below would exclude the lower mobility particles, and the sorter would select exclusively the higher mobility particles. On the other hand, at higher flow rates (milliliters per hour), the wave function generator was insufficient to provide voltages that would induce a strong enough acoustic field to force any particles into the strong stream of clean medium. For those cases, an RF amplifier (Mini-Circuits ZHL-32A+) had to be added to the system, and the sorter was operated at around 25 V_PP_.

The experiments with the influence of flow rate on the functionality of the sorter were conducted on a model mixed suspension of polystyrene microspheres, sizes of 1 and 8 μm (Videos V1 and V2). The most efficient flow rate ratio between the primary input (particle mixture) and the secondary input (fresh medium) was 1:4. Up to 1 mL/h of the particle input was easily controllable, the particles would not spontaneously enter the secondary output, and the ASW could deflect the larger particles into the 4 mL/h stream of the fresh medium. Above these flow rates, it was increasingly difficult to prevent the particles from drifting into the secondary outlet without making the stream of the fresh medium too strong for the ASW to overcome.

These operational flow rates naturally influence the overall processing throughput, making the device particularly advantageous for lab-on-a-chip platforms. An important benefit of the acoustophoretic sorter is its versatility: it can be integrated into virtually any microfluidic system, either as a built-in feature of a microfluidic chip or as a standalone module connected as an online add-on.

The application of the sorter device as a purifier of the PS-AgNPs followed the general operation procedure. The suspension of the PS-AgNPs, free NPs, and general debris was continuously flown from the primary input channel, while a fresh medium was supplied through the larger input channel at a higher flow rate. When entering the separation chamber, the stream of particles followed a straight trajectory toward the smaller primary outlet channel, which was supported by the suppressive drag of the fresh medium. Generating an acoustic field of 1.70–1.85 MHz formed the ASW in the separation chamber, which deflected PS-AgNPs toward the central node, while the much smaller debris continued toward the primary outlet, much more weakly affected by the radiation forces of the ASW. Because the separation chamber and the unevenly sized channels were designed so that the central node of the ASW overlapped at least 50 μm into the larger outlet channel, the deflected microspheres could be collected in the secondary outlet purged of the byproducts of the AgNP growth and coating procedure. The capacity of the device was demonstrated through the purification of PS-AgNPs from excess AgNPs, and the same principle can be readily applied to the purification of various types of micro-objects (e.g., bacteria or microplastics). Consequently, ASW-based purification could potentially be performed directly as part of an analytical assay.

The successful separation could be observed visually. The fluid collected at the primary outlet preserved the yellow color of the sample caused by the colloid of the free AgNPs, albeit diluted by the addition of fresh medium from the secondary input. On the other hand, the secondary output was colorless, which indicated little to no free AgNPs. After a little while, the diverted PS-AgNPs could be observed to sediment in the secondary fraction while the primary fraction stayed without sediment (Figure 9A).

**Figure 9:**
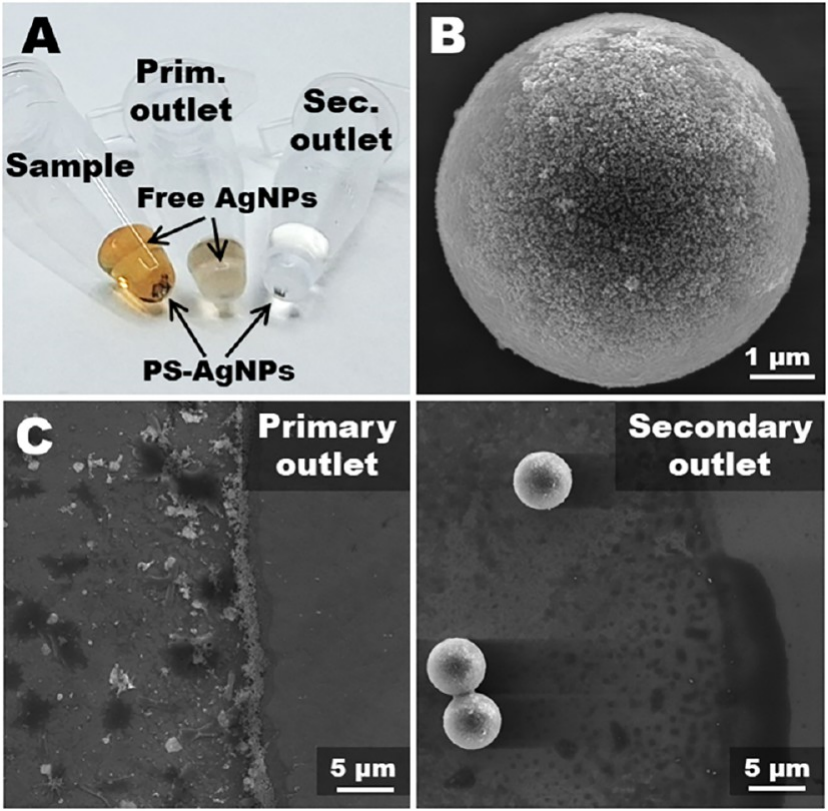
(A) Picture of collected fractions and the original sample after 1 h of sedimentation. (B) SEM image of a PS-AgNP microsphere collected in the secondary outlet. (C) SEM images of the edges of the dried spots of the fluids collected at the respective outlets.

To validate the purification process microscopically, the collected fractions were analyzed by SEM (Figures 9C,D and S12). By taking advantage of the coffee-ring effect, which concentrates nanoparticles at the periphery of an evaporated droplet, we focused our analysis on the edges of the dried spots. The images reveal a stark contrast between the two outlets. The sample from the primary (waste) outlet exhibited a dense, continuous rim formed by stacked silver nanoparticles and crystals of salts. Conversely, the fluid from the secondary (collection) outlet contained only sparse, isolated nanoparticles, confirming that their passage was successfully prevented. Additionally, the detailed figures of the surface of purified 5 μm PS microsphere show excellent preservation of the silver surface (Figure 9B). This direct confirmation underscores the high efficiency of the acoustophoretic device and validates its capability to purify PS-AgNPs composites by effectively removing the free nanoparticle fraction.

The combination of simple fabrication, effective acoustofluidic purification, and strong SERS performance positions the PS-AgNPs microspheres as a readily deployable platform for label-free chemical and biological sensing.

## 4 Conclusion

In this work, we presented a comprehensive pathway for the preparation and purification of PS-AgNPs composite microspheres, addressing key challenges that have traditionally limited their widespread application. Our approach is founded on the discovery that robust and tunable coatings of silver nanoparticles with controlled nanoscale morphology can be achieved on commercially available polystyrene microspheres (diameters ≥5 μm) through a simple coincubation process. We have demonstrated that this spontaneous attachment is mediated by residual polymeric stabilizers (namely, PVA) on the microsphere surface, a byproduct of their commercial production. This finding is significant, as it bypasses the need for laborious in situ reduction or complex surface functionalization. The method is both highly accessible and versatile, has proven effective with various types of AgNPs, and provides a straightforward means to tailor the nanoscale roughness of the silver layer by simply adjusting the AgNPs/PS ratio.

These PS-AgNPs composites were further validated as functional SERS substrates, where decorated microspheres provided clearly detectable and reproducible Raman responses using adenine and thiamine as model analytes. This demonstration confirms that the developed stabilization and purification strategy renders PS-AgNPs directly applicable to SERS-based sensing. While this synthetic route is remarkably straightforward, it inherently produces a crude mixture containing a substantial fraction of free nanoparticles. To resolve this, we developed a dedicated acoustofluidic sorting device. The principal innovation lies in its fabrication; we successfully employed isotropic wet etching of glass, a strategy that challenges the prevailing assumption that only channels with precise, rectangular cross sections achieved via expensive dry etching are suitable for effective acoustophoresis. We conclusively showed that the resulting rounded channel geometry is fully capable of sustaining a stable acoustic standing wave for highly efficient particle separation. This breakthrough significantly lowers the barrier to entry for this technology, establishing acoustofluidic purification as a far more accessible and scalable strategy.

The device effectively separates the larger PS-AgNPs composites from unbound nanoparticles based on their significant differences in acoustophysical properties, with particle size being the dominant factor. The result is a continuous, label-free, and easily achievable flow-through purification process.

In summary, this work establishes a complete methodology to produce high-purity, well-characterized PS-AgNPs composites with tailored nanosurface properties, making them immediately viable for demanding applications in fields such as catalysis, SERS-based sensing, and advanced antibacterial materials. Moreover, the validation of wet-etched glass for acoustofluidic devices opens up new possibilities for the purification of other nanomicro composite systems, offering a robust and cost-effective platform technology for researchers in materials science and beyond.

## Supplementary Material







## Data Availability

The original experimental data are available: https://doi.org/10.57680/asep.0638298.

## Notes

Additional experimental details, materials, and methods, including photographs of the experimental setup (PDF)
Operations of a microfluidic device (MP4)
Operations of a microfluidic device (MP4)

## Abbreviations

AgNPs: silver nanoparticles
ASW: acoustic standing wave
CV: coefficient of variation
LOD: limit of detection
PS: polystyrene
PVA: poly­(vinyl alcohol)
PVP: polyvinyl pyrrolidine
PZT: lead zirconium titanate
PS-AgNPs: polystyrene microparticles decorated with silver nanoparticles
SEM: scanning electron microscopy
SERS: surface-enhanced Raman spectroscopy
SLA: stereolithography
SPLITT: split-flow thin
