# Supplementary material for: Streamlined Fabrication and Acoustofluidic Purification of Silver-Decorated Polystyrene Microspheres (PS-AgNPs) for SERS Applications
Source: ACS Appl Nano Mater. 2025 Dec 30;9(1):721–32. doi: 10.1021/acsanm.5c04981 (PMC12797264; doi:10.1021/acsanm.5c04981)
Supplement: Supplementary file 1 [file an5c04981_si_001.pdf]

# Supporting Information

## Streamlined Fabrication and Acoustofluidic Purification of Silver-Decorated Polystyrene Microspheres (PS-AgNPs) for SERS Applications

*Jakub Novotny[1,$], Lucie Brezinova[1,2], Vit Pavelka[1], Anna Tycova[1,$,*]*

[1]Institute of Analytical Chemistry of the CAS, v.v.i., Brno, 602 00, Czech Republic
[2]Department of Chemistry, Masaryk University, Brno, 625 00, Czech Republic
*tycova@iach.cz

[$] J.N. and A.T. contributed equally to this paper.

### Purification of PS-AgNPs from free and poorly attached AgNPs

The PS-AgNPs were prepared as described in the text of the manuscript. For this experiment, the synthesis operated with 5× concentrated colloid (i.e., 135 µg/mL of silver).

The PS-AgNPs dispersion was after the synthesis purified using centrifugation. The 1 000 µL of dispersion was centrifuged for 1 minutes at 2 000 RCF (Mikro 22, Hettich). The volume of 800 µL supernatant was discarded and replaced with fresh deionized water. The process of centrifugation was repeated three times in total, thus the percentage of original liquid dropped down to 0.8%. This highly purified dispersion was stored at laboratory temperature for 12 days. The representative microspheres in various steps of the process of the purification are shown in Figure S1.

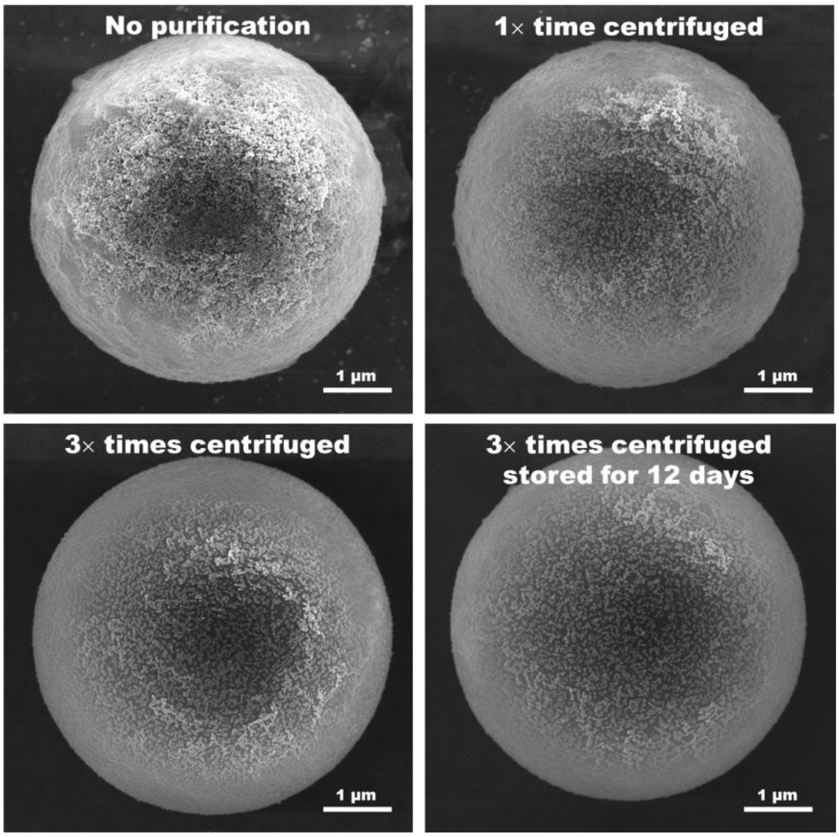


Figure S1. SEM figures showing the effect of centrifugation and storage on the surface of PS-AgNPs.

## The surface morphology of PS microspheres

From their chemical behavior, we concluded that the growth of the commercially available microspheres with the diameter <5 µm is controlled by surfactants (i.e., micelles microreactors), while the larger diameters rely on the polymer-based stabilization. Detailed SEM and AFM analyses of the bare PS surfaces indeed revealed differences in their surface characteristics (Figure S2). While the 2-µm PS microspheres (solid content 2%, Supelco) exhibited a perfectly smooth surface, the 5-µm microspheres (solid content 10%, Supelco) displayed a textured surface. We attribute these differences to variations in the synthetic pathway.

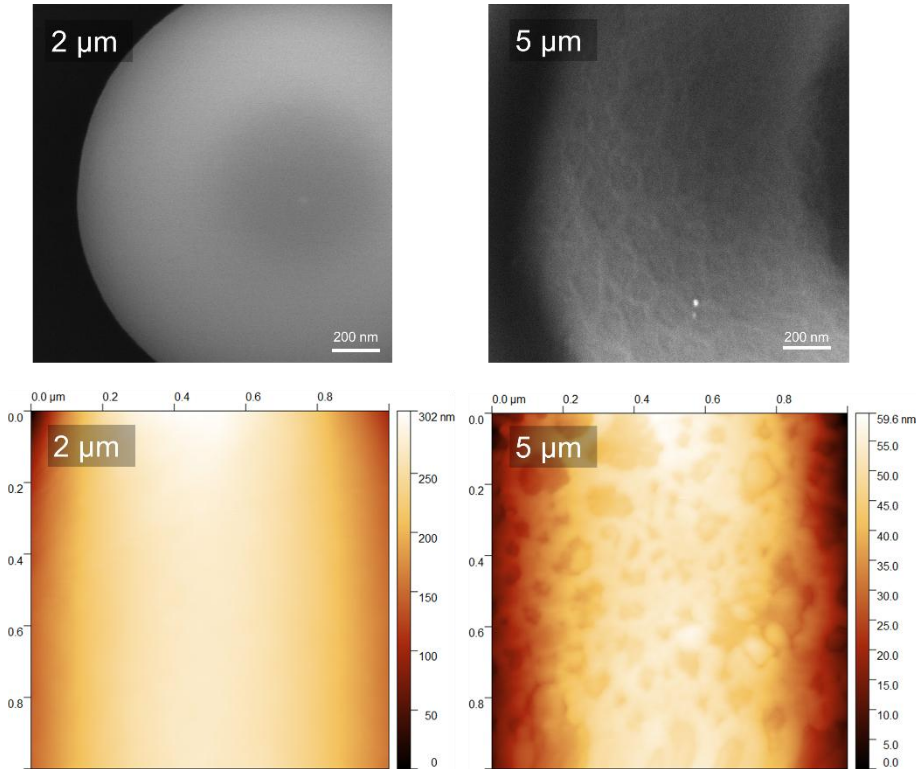


Figure S2. Detailed microscopic figures of bare PS microspheres with a diameter of 2 µm and 5 µm, respectively. Up – SEM images. Down – AFM images. AFM image were acquired using a *Bruker AFM Icon* in tapping mode with an ACM tip (tip diameter ≈ 6 nm).

## SERS-based characterization of polystyrene microspheres

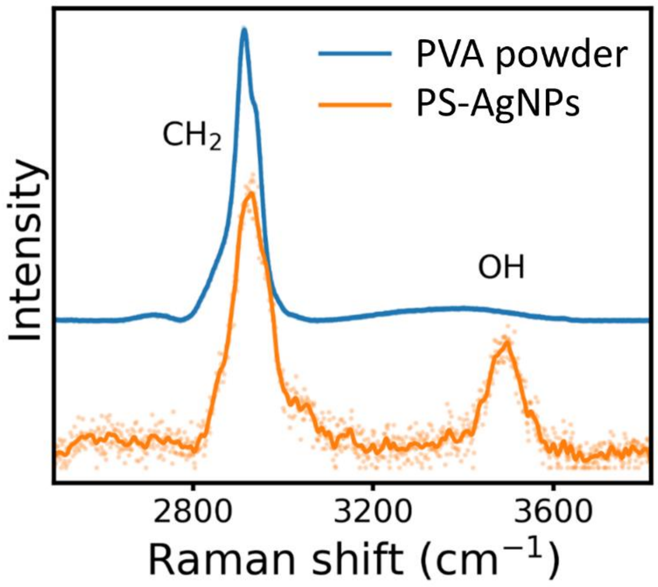


Figure S3. $CH_2$ and OH characteristic vibrational transitions of PVA acquired (blue) conventionally from powder and (orange) via SERS mapping experiment of purified PS-AgNPs microspheres dried on a silicon wafer. 10 spectra with the most prominent OH signal were automatically chosen and summated. Spectra are normalized.

## Effect of polyvinyl alcohol (PVA) solution on the PS-AgNPs synthesis

We investigated the effect of spontaneous AgNPs immobilization with respect to the size of PS microsphere (the production procedure, respectively). We run identical experiment as described in the main text, however, we used the PS microspheres from different producers:

10 µm (solid content 10%, Microparticles)
1 µm (solid content 10%, Sigma Aldrich)
0.5 µm (solid content 2.7%, Polysciences)

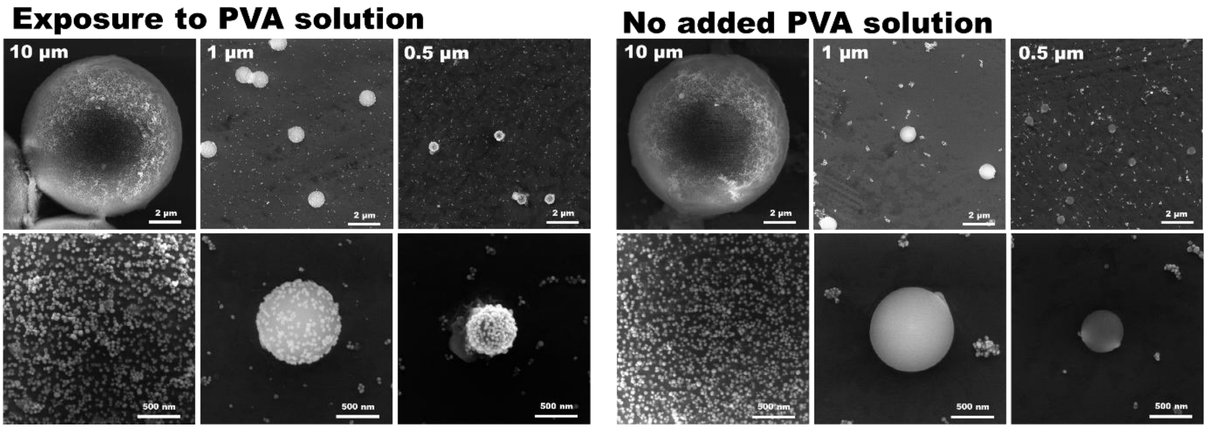


Figure S4. SEM figures of polystyrene microspheres (diameters in the range of 0.5, 1 and 10 µm) after co-incubation with AgNPs. The figure shows the effect of PVA solution on the surface coverage of PS microspheres.

The observations graphically summarized in Figure S4 are in absolute agreement with the results given in Figure 5. This strongly support our hypothesis and shows applicably of our synthetic pathway to broadly available products.

## The effect of in-situ and decoupled reduction of AgNPs on the homogeneity of PS-AgNPs

In this experiment, we focused on the effect of increased concentration of the reactants (needed for formation of AgNPs) in the presence of PS microspheres with the special attention on the homogeneity of the PS-AgNPs dispersion. The procedure followed the same protocol as described in the main text of the manuscript – i.e., silver cations were reduced by hydroxylamine hydrochloride at basic conditions. The PS microspheres were purified by centrifugation prior to their use.

As we worked with the fixed reaction volume, we added the same volume of reactants and changed the concentration of their solutions – see the Table S1. The pH values of the reaction mixtures were in the range of 10.0-11.5.

Table S1. The mass concentrations of reactants used for in-situ synthesis of PS-AgNPs. The reaction volume remained constant.

| Concentration factor of Ag | NaOH (58 µL) | Hydroxylamine hydrochloride (58 µL) | DI water (317.5 µL) | 5 µm PS sphere (5 µL) | $AgNO_3$ (61.5 µL) |
|---|---|---|---|---|---|
| no Ag | | | | Solid content 5% | |
| 1× | 0.96 mg/mL | 0.42 mg/mL | | Solid content 5% | 0.34 mg/mL |
| 2.5× | 2.40 mg/mL | 1.04 mg/mL | | Solid content 5% | 0.85 mg/mL |
| 5× | 4.80 mg/mL | 2.08 mg/mL | | Solid content 5% | 1.70 mg/mL |
| 10× | 9.60 mg/mL | 4.15 mg/mL | | Solid content 5% | 3.40 mg/mL |

The SEM figures show that the surface coverage of AgNPs on the surface of PS is not significantly increased and is comparable for 70-270 µg/mL silver concentration. The SEM pictures with lower magnification reveals that the dispersions exhibit poor stability. Indeed, observing the sedimentation speed, we noticed that the PS-AgNPs prepared in-situ, sedimented faster and the dark color of the sediment suggested the formation of massive clusters.

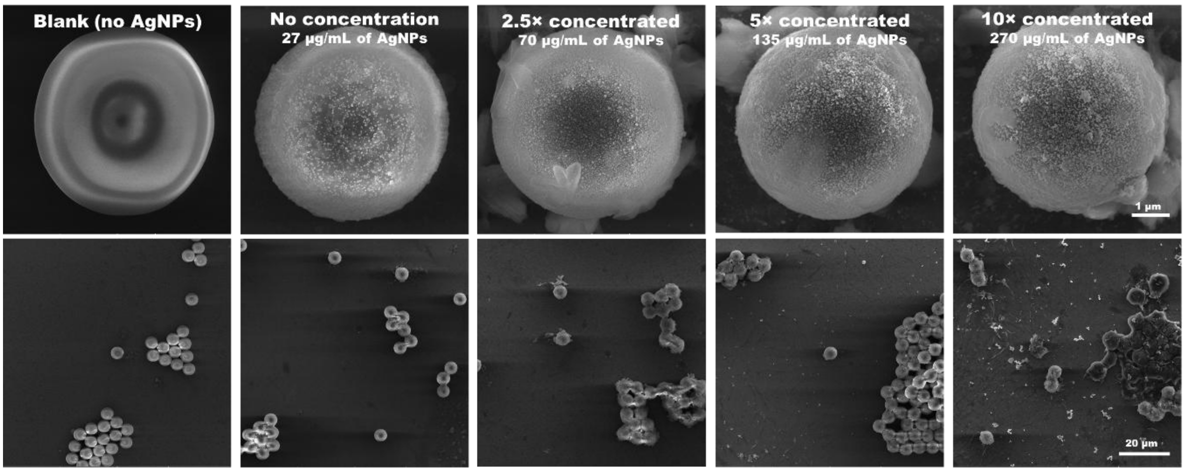


Figure S5. SEM figures depicting: Up – the change in the density of AgNPs on the surface of PS microspheres. Down – clustering of the dispersion.

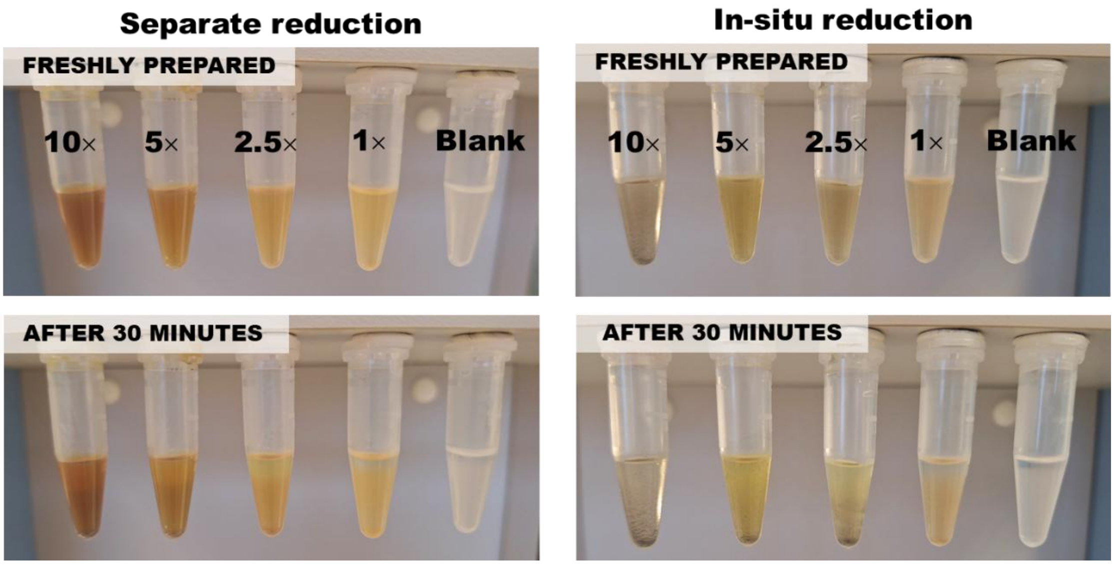


Figure S6. The comparison of the sedimentation speed of PS-AgNPs dispersion if the reduction of AgNPs occurred separately or in-situ.

To understand the process of clustering, we synthesized silver colloids using concentrations in Table S1. However, we did not introduce any PS microspheres. Thus, the colloid was purely based on AgNPs. All four obtained colloids were diluted by water to obtain equal concentration of silver (i.e., 5.5 μg/mL) and were measured their absorption spectra by UV-Vis spectrometer (Shimadzu 1800).

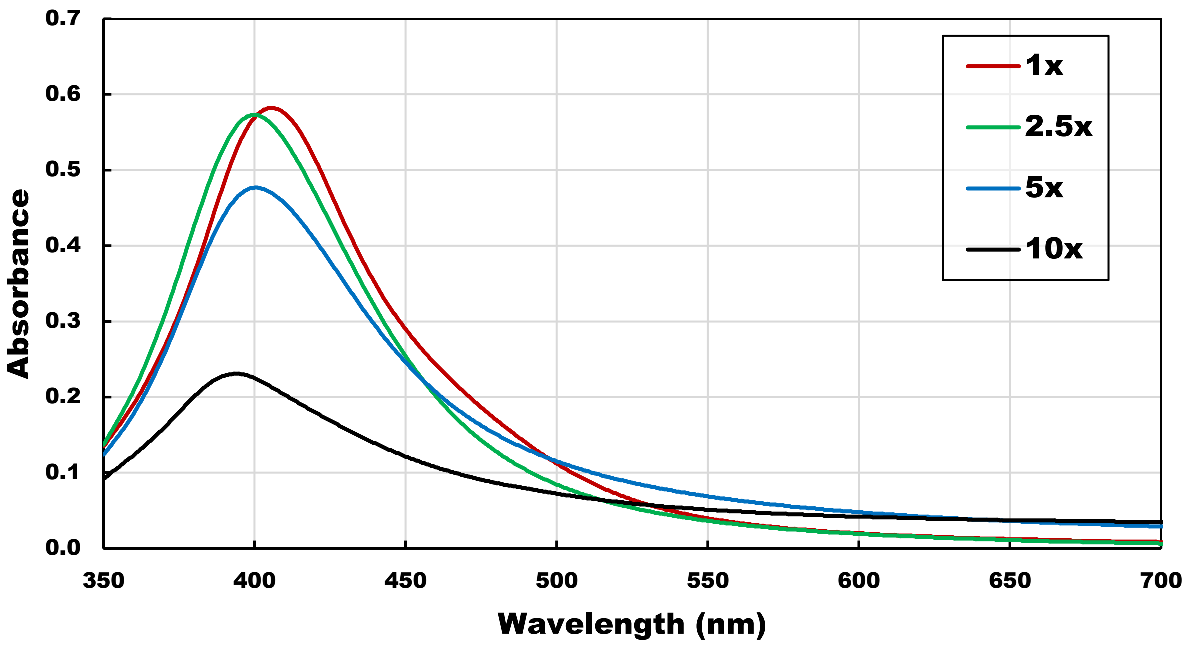


Figure S7. UV-Vis absorption spectra of silver colloids synthesized by the reduction of hydroxylamine hydrochloride using various reactant concentrations – see Table S1.

## Protocol for the synthesis of silver nanoparticles (AgNPs)

We synthesized various types of silver nanoparticles using different widely spread protocols. To reach stable conditions, all the colloids were left to ripen at least 24 hours before their use. All the reactions were based on the reduction of silver cations from silver nitrate using various reductants:

(1) Sodium borohydride:[1] The volume of 30 mL of 3 mM $NaBH_4$ was cooled down by an ice bath for 20 minutes. Then, 2 mL of silver nitrate was dropwise added to this solution at constant stirring. The colloid was left next 30 minutes in an ice bath and finally stored in the fridge. The concentration of silver in the colloid was 6.7 µg/mL.

(2) Sodium citrate:[2] To 150 mL of water heated to boiling in an oil bath under a reflux condenser, 4.8 mL of 20 mM sodium citrate tribasic dihydrate and 3.0 mL of 55 mM silver nitrate were added with continuous stirring. The reaction mixture was kept at boiling for 90 minutes without direct light exposure. The concentration of silver in the colloid was 112 µg/mL. The colloid was stored at laboratory temperature.

The nanoparticles were concentrated via microcentrifuge (MiniSpin Plus, Eppendorf) using 10 000 RCF. The time of centrifugation was influenced by their size. For AgNPs reduced by $NaBH_4$ the time was set to 12 minutes and for AgNPs reduced by citrate to 3 minutes. Various volumes were discarded to obtain the desired concentration of silver. The sediment with residual supernatant was homogenized by 5-10 seconds of sonication and short vortexing.

## The interaction of AgNPs reduced by various reduction agents with PS surface

The volume of 10 µL of purified PS microspheres were mixed with 990 µL of AgNPs. The AgNPs did not undergo any special chemical pretreatment but the concentration by centrifugation. The mixture of PS and AgNPs was continuously mixed for 5 minutes. Finally, the dispersion was stored at least for 30 minutes at laboratory temperature prior to its characterization.

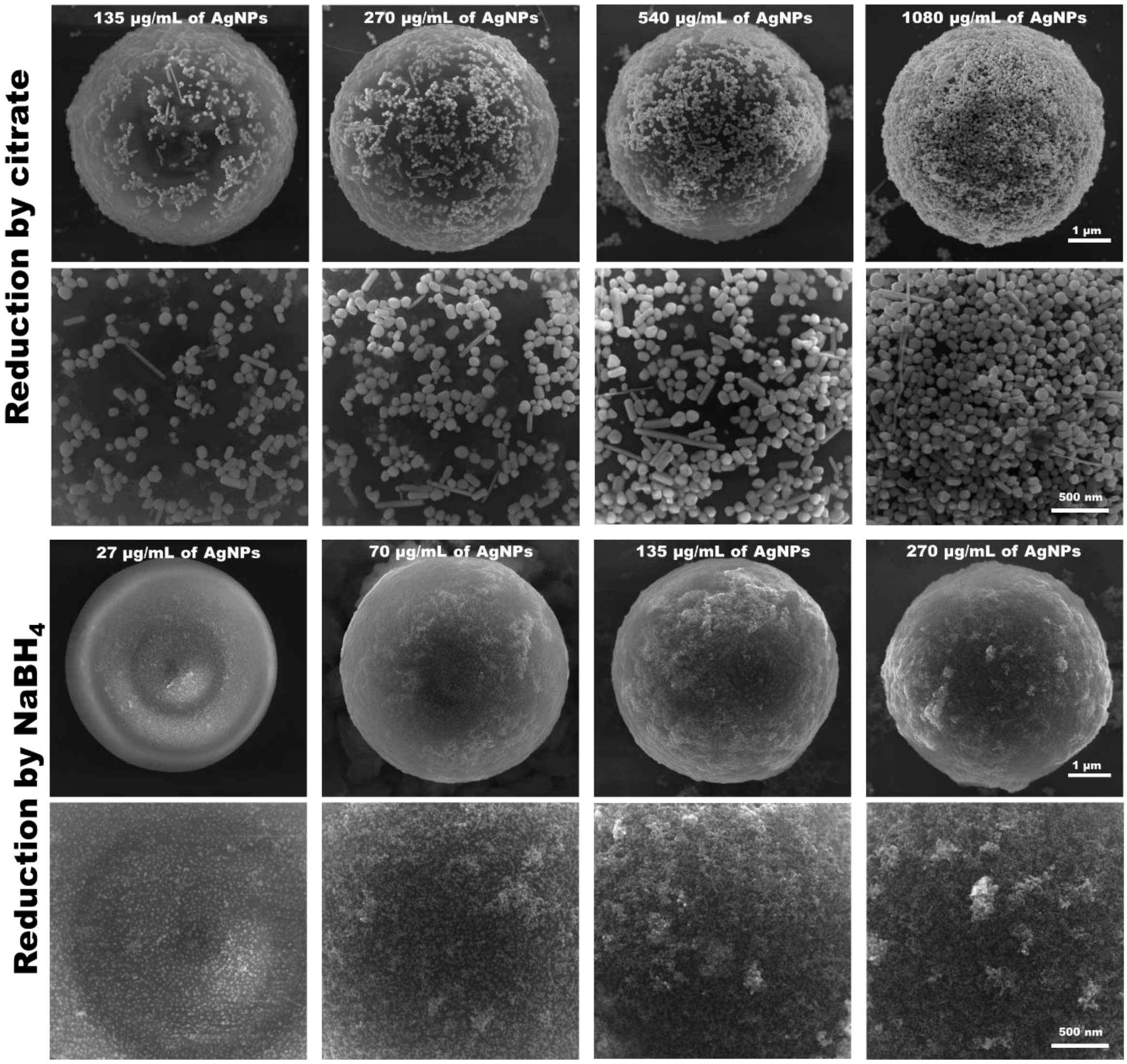


Figure S8. SEM figures depicting the change in the density of AgNPs on the surface of the polystyrene microspheres (diameter of 5 µm) in two various magnifications. The nanoparticles were reduced separately by: up - sodium citrate, down – sodium tetrahydroboride.

## Geometry of wet etched-channels

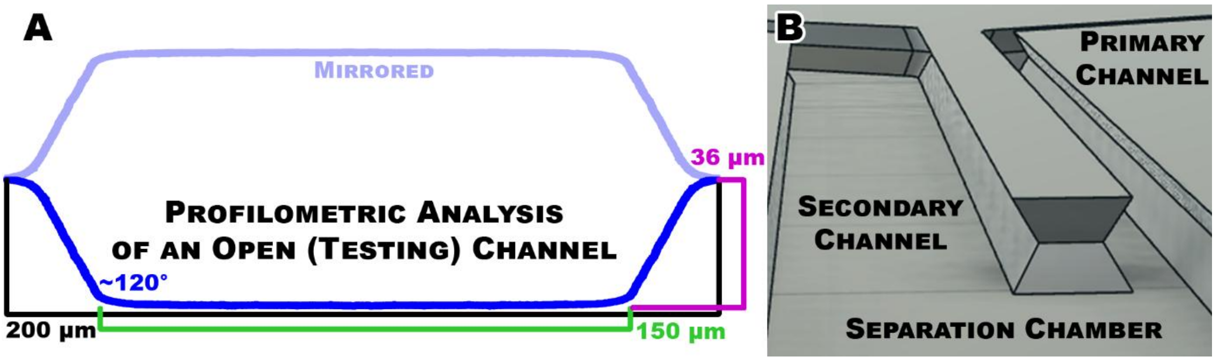


Figure S9. A: Visualization of the profilometric analysis of a testing microchannel (with stylus profilometer KLA/TENCOR AlphaStep). The formation of ASWs was initially observed in a microchannel of the same design. B: CGI approximation of the sorter junction based on the profilometric analysis. The sorter was fabricated according to the identical procedure as the testing microchannel.

## Optimization of chip layout

In the initial design with the inlet and outlet openings situated on the surface of the chip, these had to be drilled into one of the glass pieces using a diamond dental burr. Unfortunately, this design had to be dropped in favor of the side openings because of the issues with clogging. The main contributors were the size difference from the relatively large drilled opening into the hair-thin etched microchannel, which caused accumulation of particles before the transition. This issue was amplified because the trajectory of the flow made a sharp 90° turn from the inlet opening into the channel. The design was then changed to a smaller chip with in/outlets on the sides.

For our initial chips with drilled inlets, a 3D-printed latch-on case fitted with the fluidic connectors was designed to allow quick disassembly of the fluidic connections and the release of the accumulated particles. The PEEK capillary fittings (LabSmith) or Luer lock connectors could be screwed into the 3D-printed case and the connection was sealed by small o-rings compressed around the surface of the input openings in the glass device by the system of latches (Figure S10).

Compared to the initial "top-loader" design, the slotted holders for the cut-out chips with the side in-/outlets were much easier to design since each chip could be cut to identical external dimensions. One of the effects of that was that the holder could be designed much smaller. The holder for "top-loader" chips had to be designed quite sturdy to withstand the forces of the latch-on system and therefore consumed over 120 mL of the resin over all the components of the holder. On the other hand, the slotted holder for the cut-out chips with screw in fluidic connectors, required 7 mL of resin + 5 mL for printing supports, for 12 mL overall consumption, making it a 90% reduction in material costs.

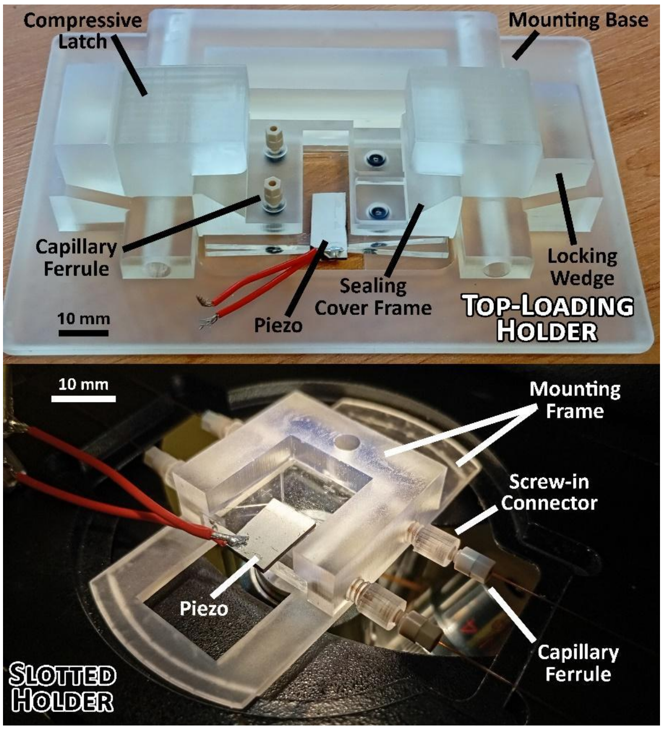


Figure S10. Comparison of the "top-loader" and the slotted chip holder. To compensate for the mechanical stress and to fit the holder into the microscope mount, the top-loader holder used up 10× the printing resin.

As an interesting note, the shape of the divider between the primary and secondary inlets and/or outlets can have significant influence on the acoustic field. The initial design featured a divider with rectangular layout (Figure S11), but a concern materialized about the risk of particles getting trapped

on the flat side of the divider perpendicular to the flow. A layout with rounded corners was tested that should have potentially allow particles to slide along the curved edge and reduce the risk of trapping. Unfortunately, a curious phenomenon emerged during the experiment with a model sample of fluorescent PS microspheres in fluorescein-dyed demi-water. While applying the acoustic field, a noticeable turbulence in the flow could be observed, pattern of which was very frequency-dependent. In the previous experiments with the rectangular divider, the laminar parallel streams of the dyed sample and the non-dyed fresh medium were mostly independent on the acoustic field.

With the rounded divider, the turbulent flow mixed the microspheres and the dye into the fresh medium. The phenomenon was observed across several chips featuring this rounded layout, and could not therefore be explained by manufacturing defects or deficiencies introduced during the assembly or operation of the microfluidic system, such as obstacles (bubbles, foreign objects, etc.) or misalignments. With the available equipment, the principle behind the disturbance could not be determined. According to one proposed explanation, the reflection of the soundwaves on the introduced curved edge could have interfered with the acoustic field in the separation chamber forming patterns that disturbed the flow of fluids. According to another hypothesis, based on the resemblance of the rounded divider to, so-called, microneedle acoustic mixers from various publications (such as Tian[3]), the rounding of the tip of the divider could have turned it into a generator of acoustic streaming. No matter the principle behind the turbulences, the design of the divider had to be returned to the rectangular layout.

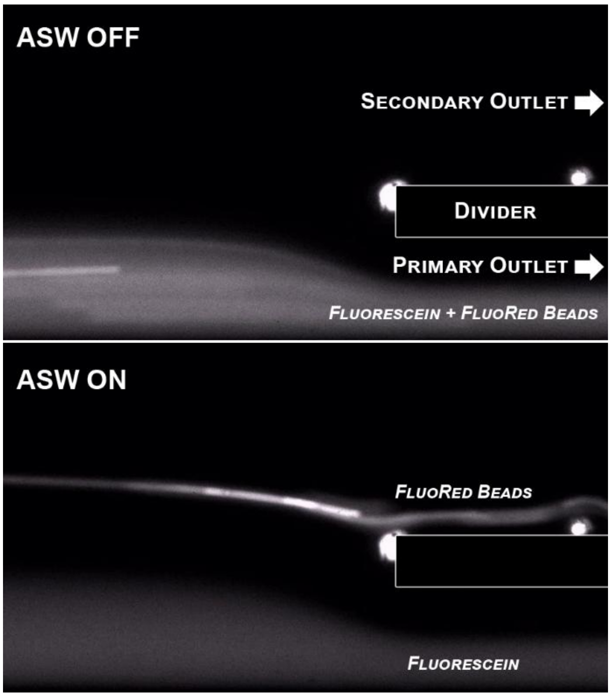


Figure S11. Visualization of the device functionality demonstration enhanced in a fluorescent system. Up - The stream of the sample mixture was visualized with the fluorescent dye. Bottom - The fluorescent microspheres were separated from the dyed medium.

## Efficiency of the acoustofluidic sorter

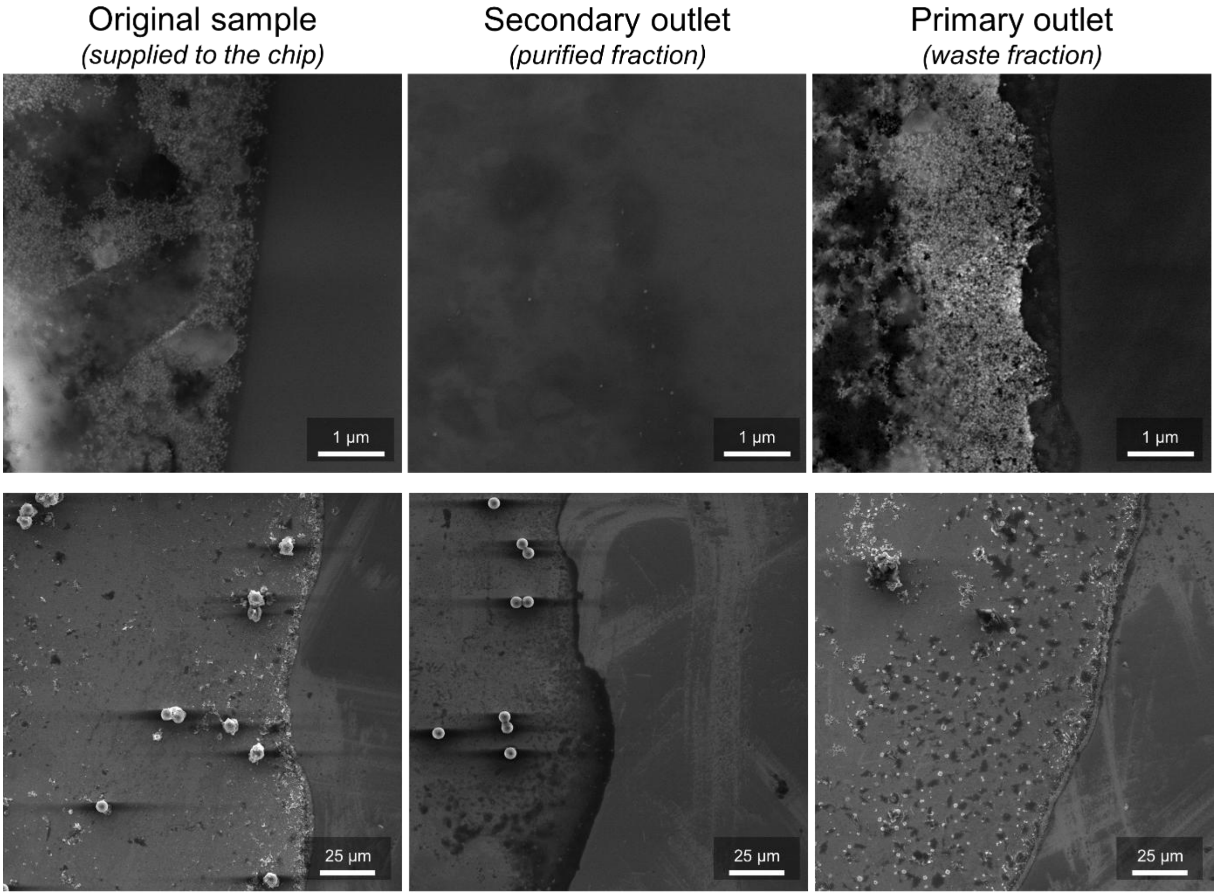


Figure S12. SEM images comparing the edges of the dried spots of the original mixture with the fluids collected at the respective outlets at two various magnifications.

## References


(1) Creighton, J.; Blatchford, C.; Albrecht, M. Plasma Resonance Enhancement of Raman-Scattering by Pyridine Adsorbed on Silver or Gold Sol Particles of Size Comparable to the Excitation Wavelength. *Journal of the Chemical Society-Faraday Transactions Ii* **1979**, *75*, 790–798. https://doi.org/10.1039/f29797500790.

(2) Lee, P.; Meisel, D. Adsorption and Surface-Enhanced Raman of Dyes on Silver and Gold Sols. *J. Phys. Chem.* **1982**, *86* (17), 3391–3395. https://doi.org/10.1021/j100214a025.

(3) Tian, C.; Liu, W.; Zhao, R.; Li, T.; Xu, J.; Chen, S.-W.; Wang, J. Acoustofluidics-Based Enzymatic Constant Determination by Rapid and Stable in Situ Mixing. *Sensors and Actuators B: Chemical* **2018**, *272*, 494–501. https://doi.org/10.1016/j.snb.2018.05.149.
